# Function and Structure Relationships With Inflammation Differ in Two Chronic Suppurative Lung Diseases

**DOI:** 10.1002/ppul.71659

**Published:** 2026-05-08

**Authors:** Teresa Fuchs, Jacqueline Donovan, Andrew Ives, Samantha Irving, Gwyneth Davies, Claire Hogg, Eric Alton, Gemma Wilson, Andrew Bush, Jane C. Davies

**Affiliations:** ^1^ National Heart & Lung Institute, Imperial College London London UK; ^2^ Imperial Biomedical Research Centre London UK; ^3^ Royal Brompton Hospital, Guy's & St Thomas' Trust London UK; ^4^ Department of Child and Adolescent Health Paediatrics III, Medical University of Innsbruck, Cystic Fibrosis Centre Innsbruck Austria; ^5^ Population Policy and Practice, UCL Great Ormond Street Institute of Child Health London UK

**Keywords:** cystic fibrosis, inflammation, long‐term, primary ciliary dyskinesia, sputum

## Abstract

**Rationale:**

Cystic fibrosis (CF) and primary ciliary dyskinesia (PCD) are characterized by neutrophilic airway inflammation but differ in clinical features.

**Objectives:**

We investigated relationships of pulmonary and systemic inflammatory markers with functional and structural lung disease.

**Methods:**

Systemic (CRP, IgG, IL‐6, and IL‐8) and sputum (calprotectin, IL‐6, and IL‐8) markers were measured at baseline. Relationships were explored with spirometry, lung clearance index and lung computed tomography (CT) scores at baseline and with spirometry 10 years later.

**Results:**

42 patients (21 CF, 21 PCD) of similar age (median CF 27 y [12–59], PCD 27 y [11–62]) and lung function (median ppFEV_1_CF 59% [40–99], PCD 67% [30–101]) were enrolled in 2009. Systemic inflammation was significantly higher in CF (Neutrophils, *p* < 0.05, IL‐6 and 8, *p* < 0.01). Conversely, sputum IL‐6 and 8 were higher in PCD (*p* < 0.01). In CF, sputum IL‐6 counter‐intuitively correlated with better CT scores (*r* = −0.51/*p* < 0.05) at baseline and with better spirometry (*r* = 0.58, *p* < 0.05) 10 years later. Conversely, high sputum IL‐6 at baseline was associated with worse lung function in PCD (*r* = 0.81/*p* = 0.001) after 10 years.

**Conclusion:**

This study emphasizes the differences of functional and structural aspects in both diseases. Markers that predict long‐term outcome in PCD and in CF were identified. The most striking and unanticipated finding was that sputum IL‐6 correlated with better CT scores and lung function in CF. This paradox merits further research but challenges whether sputum IL‐6 in CF is always a bad prognostic indicator.

**Abbreviations**
AS: airway surface
CF: cystic fibrosis
CRP: c‐reactive protein
CT: computer tomography
ETI: elexacaftor/tezacaftor/ivacaftor
FEV1: forced expiratory volume in the first second
FVC: forced vital capacity
IgG: immunoglobulin G
IL: interleukins
LCI: Lung Clearance Index
MCC: mucociliary clearance
NE: neutrophil elastase
PCD: primary ciliary dyskinesia
WCC: white cell count

## 1 Introduction

Cystic fibrosis (CF) is a single gene disorder characterized by loss of function of the CF transmembrane conductance regulator protein, a chloride and bicarbonate channel expressed in the apical membrane of multiple epithelial cells [1]. In the lungs, thick mucus and airway surface (AS) dehydration impair mucociliary clearance (MCC). Abnormal AS pH has also been described in CF animals leading to impaired function of pH sensitive antimicrobial host defences. Early onset inflammation and recurrent infections result in progressive lung damage, detectable on lung function testing and computer tomography (CT) scanning [1].

Primary ciliary dyskinesia (PCD) is predominantly inherited as an autosomal recessive condition; mutations have been described in multiple genes encoding structural ciliary and assembly proteins resulting in impaired MCC [2]. In PCD, diagnosis is often delayed, causing repeated infections including sinusitis, otitis media and bronchitis. Clinical manifestations in PCD typically include onset of respiratory symptoms at birth with an early onset of a productive cough and rhinitis [3].

Thus, despite different mechanisms of impaired MCC and mucus accumulation, pathophysiological consequences are similar with recurrent infections and chronic inflammation in both diseases. However, clinical outcomes differ [4]. Understanding the factors that favor PCD patients is less clear. Better cough clearance and differences in the age of onset of bronchopulmonary pathogens such as *Staphyloccocus aureus* and *Pseudomonas aeruginosa* have been proposed [5]. There are also significant differences in the inflammatory processes between the diseases, but both are characterized by neutrophil dominated airway inflammation [6, 7]. In CF, pathologically altered neutrophils release excessive amounts of granule contents including neutrophil elastase (NE) [6]. Increased concentrations of other pro‐inflammatory mediators such as interleukin (IL)‐1β, IL‐6, or IL‐8 are also found in the CF airways [8]. Inflammation in PCD is also strongly associated with IL‐8 concentrations and airway neutrophilia [9]. However, the ciliary abnormalities in PCD do not appear to lead to the same degree of inflammation as in CF. Comparison of inflammatory markers in sputum in patients with CF and PCD during pulmonary exacerbations showed higher values in PCD [10]. Analyzing the same markers in response to treatment of pulmonary exacerbations also showed a diverse pattern between the diseases indicating differences in airway inflammation [10]. Another study has shown no difference between biophysical properties of sputum between the two diseases; however, IL‐8 was found to be higher in PCD [9].

The primary aim of this study was to compare systemic (serum) and local (airway lumen) inflammatory markers in people with CF and PCD and to investigate relationships with functional and structural measures of lung disease. We opportunistically looked at a limited dataset after a 10‐year period to further interrogate relationships observed. We hypothesized that there would be differences in the relationship between inflammatory markers and prognosis between these two diseases.

## 2 Methods

### 2.1 Study Population

Age and sex matched patients diagnosed with CF and PCD on standard criteria [11, 12] treated at the Royal Brompton Hospital, London were recruited for the study. Participants were included according to the following criteria: >10 years of age, forced expiratory volume in the first second (FEV_1_) ≥ 40%, and clinical stability for at least 1 week prior to the study visit. Pulmonary chronic infection with *Burkholderia cepacia complex*, chronic oxygen requirement, lung transplant, and pregnancy were exclusion criteria. Local and systemic inflammatory markers, chest CT scans, multiple breath washout, and spirometry were performed at baseline. Long‐term follow‐up analysis included correlation of baseline inflammatory markers with spirometry 10 years later. Lung function data in 2019 was collected retrospectively as part of the annual reviews. Clinical stability was assessed based on medical history. From the original CF cohort, four patients were lost to follow‐up and two died. We excluded one further subject who had been prescribed ivacaftor, none of the remaining CF patients received dual‐combination CFTR modulator therapy in 2019. From the original PCD cohort, six were lost to follow up and two died. Thus, 14 patients with CF and 13 with PCD were included in this analysis. The study was approved by local ethics committee (National Research Ethics Service, Kings College Hospital Research Ethics Committee, REC reference 07/Q0703/78; 2007).

### 2.2 Cytokine Analysis

Systemic markers analyzed were white cell count (WCC), absolute neutrophil count, C‐reactive protein (CRP), Immunoglobulin G (IgG), serum calprotectin, and IL‐6, and 8. WCC, neutrophils, IgG and CRP were measured as part of clinical assessment in the clinical laboratory. Detailed information regarding cytokine measurements is shown in the online supplement. Sputum was obtained by spontaneous expectoration and processed for analysis of total cell count, IL‐6, 8 and 1β, NE, and sputum calprotectin. In four patients (one with PCD, three with CF) sputum was induced; however, due to the small number, these samples were not analyzed separately. Details of the assay kits and their lower limits of detection are described in the online supplement (Table E8).

### 2.3 Assessment of structural and functional lung disease

The performance of spirometry and multiple breath washout measurements are described in more detail in the online supplement. Chest CT imaging was scored on a lobar basis by two independent radiologists using a grading system based on Roberts et al. [13]. The following features were assessed in the chest CT: overall score and total bronchiectasis score, extent of bronchiectasis, wall thickness, small and large plugs as well as air trapping (see online supplement).

### 2.4 Statistical Analysis

Normality testing was performed using the Kolmogorov‐Smirnov test. Depending on distribution, groups at baseline were compared using T‐test or Mann‐Whitney *U* test. Values were corrected for multiple comparisons using Bonferroni correction. The Mann‐Whitney *U* test was used for comparison with the follow‐up visits. A paired T‐test was used for longitudinal comparison within the groups. Correlations were performed using the Spearman non‐parametric correlation. The study was exploratory and hypothesis generating; correlations were not corrected for multiplicity and p values should be considered nominal. Data are expressed as median and range and the null hypothesis rejected at *p* < 0.05. Statistical analysis was performed with IBM SPSS, version 29.0 (IBM, Ehningen, Germany) and Prism, version 9.5.1 (GraphPad Software Inc. Boston, U.S.A).

## 3 Results

### 3.1 Baseline Patient Demographics

Forty‐two people, 21 each with CF and PCD, contributed baseline data, samples for inflammatory markers and CT scans (Table 1). While age and gender were deliberately matched between disease groups, FEV_1_ percent predicted and Lung Clearance Index (LCI) serendipitously also showed comparable values. The LCI was abnormal (>7.4) for every participant in both groups and most of the patients showed reduced FEV_1_% (median FEV_1_% 59.3 in CF and 67.1 in PCD, not significant). For CT scans, median total disease score and bronchiectasis score were significantly greater in CF, with more severe structural lung damage compared to PCD (*p* < 0.001). Ten patients with CF and seven with PCD were considered as chronically infected with *P. aeruginosa* (*p* < 0.05) [15].

**Table 1:** Demographic data and differences of study groups at baseline 2009.

Variable	CF	PCD	*p*‐Value
Number	21	21	
Age, (y) median (range)	27 (12–59)	27 (11–62)	ns^a^
Sex, *n*			ns^a^
Male (%)	11 (52.4)	11 (52.4)	
Female (%)	10 (47.6)	10 (47.6)	
FEV_1_%, median (range)	59.3 (39.7–98.9)	67.1 (29.5–101.2)	ns^b^
LCI, median (range)	12.6 (10.4–19)	10.9 (7.6–18.7)	ns^b^
CT total score, median (range)	53.7 (24–70)	31.5 (8.8–63)	**< 0.001** a
CT bronchiectasis score, median (range)	34.5 (17.5–41.5)	18 (0–37.5)	**< 0.001** a
Chronic *P. aeruginosa*, *n* (%) [14]	10 (47.6)	7 (33.4)	**0.031** c
Azithromycin therapy, *n* (%)	14 (66.7)	2 (9.5)	**< 0.001** c
**CF genetics**			
F508del homozygote		9	
F508del heterozygote		11	
other		1	
**PCD defects**			
Outer dynein arm defect		13	
Combined outer and inner dynein arm defect		4	
Microtubular disorganization and inner dynein arm defect		2	
Microtubular transposition defect		2	

*Note:* Data is presented as median (range) or percentage. ns, not significant.

^a^ Mann Whitney Test.

^b^ Unpaired *T*‐Test.

^c^ Chi‐Square Test.

### 3.2 Cross Sectional Comparison of Inflammatory Markers Between Groups

**Figure 1:**
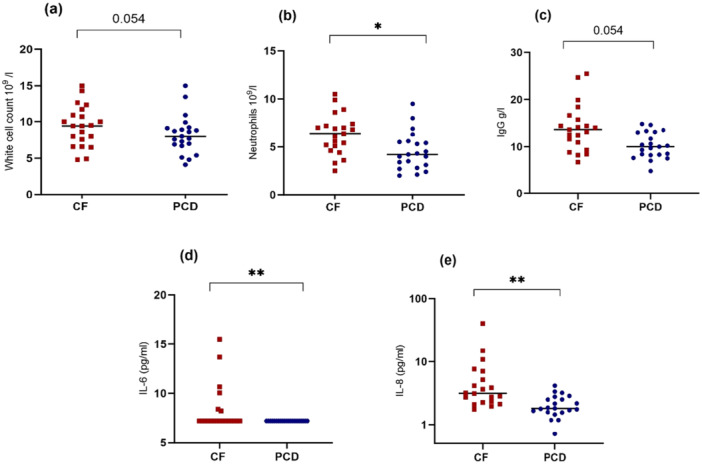
Comparison of systemic inflammatory markers between CF and PCD. Serum WCC (a)^1^, neutrophils (b)^1^, and IgG (c)^2^ show higher values in CF (red squares) compared to PCD (blue circles). IL‐6 (d)^1^ and IL‐8 (e)^1^ are significantly higher in CF. Line represents median and *p*‐value represent results of ^1^ Mann‐Whitney *U* test or^2^ *T*‐test. Results were corrected for multiple comparison using Bonferoni correction. *p < 0.05, **p < 0.01. [Color figure can be viewed at wileyonlinelibrary.com]

Comparison of *systemic* inflammatory markers measured in serum between the two disease groups showed overall higher values in CF. Significant differences were seen in neutrophil number (*p* < 0.05) and IL‐8 (*p* < 0.01). WCC and IgG were higher in CF, however not significantly. Serum IL‐6 was undetectable in all PCD patients but was detectable in CF patients (*p* < 0.01) (Figure 1 and Table E1).

**Figure 2:**
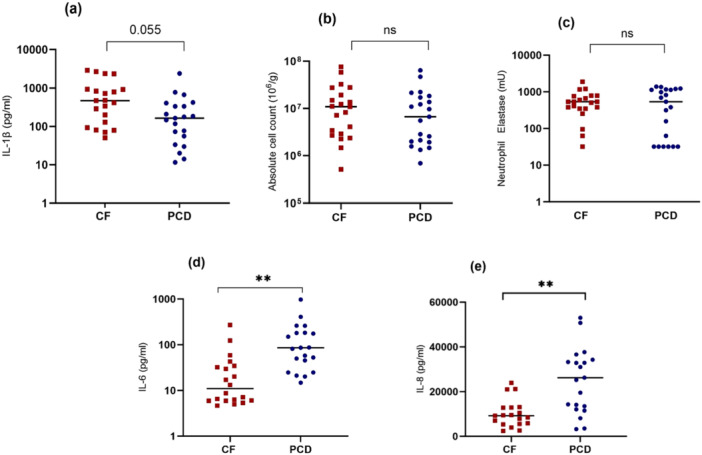
Comparison of sputum inflammatory markers between CF and PCD. Sputum IL‐1β (a)^1^, absolute cell count (b)^1^, NE (c)^2^, IL‐6 (d)^1^, and IL‐8 (e)^1^ in CF (red squares) and PCD (blue circles) patients show significantly higher values in PCD for IL‐6 and IL‐8. Line represents median and *p*‐value represent results of^1^ Mann‐Whitney *U* test or^2^ *T*‐test. One high outlier for each IL‐6 and IL‐8 have been removed in PCD in the figure. Results were corrected for multiple comparison using Bonferoni correction. **p < 0.01. [Color figure can be viewed at wileyonlinelibrary.com]

Inflammatory markers measured in *sputum* showed a more variable pattern: IL‐1β, absolute cell count, NE and calprotectin did not differ between groups. IL‐6 (*p* < 0.01) and IL‐8 (*p* < 0.01) were significantly higher in PCD sputum (Figure 2 and Table E2).

### 3.3 Assessing Relationships Between Inflammatory Markers Within and Between Compartments

To further explore disease‐related differences, we assessed the relationships between inflammatory markers and whether these relationships were different for CF and PCD. In the *serum* (Table E3), significant correlations were observed for total WBC with neutrophil number and with calprotectin, with similar significance in the two disease groups. Neutrophil count correlated significantly with calprotectin and CRP only in the PCD group. IL‐6 correlated with CRP and IgG in CF but could not be assessed in PCD due to being below the levels of detection in all patients.

In the *sputum* (Table E4), NE was significantly correlated with calprotectin in a similar way in both disease groups. A number of other markers demonstrated correlations only in one disease group and not the other. Most notable in CF, total cell count correlated with NE and calprotectin⊡ In PCD on the other hand, total cell count correlated with IL‐8 and IL‐1β. IL‐6, the outlier in our cross‐sectional disease comparison, was again an outlier here, correlating with no other marker in either disease group.

Investigating relationships *between compartments* (Table E5), three markers were measured in both sample types (IL‐6, IL‐8 and calprotectin); none of them correlated across sample type in either disease group. Sputum IL‐8 was the only lung marker relating to systemic inflammation (total WBC only) in both disease groups. Sputum NE related more broadly to systemic inflammation, but only in PCD (calprotectin, CRP, and WBC). Thus, in general, the two compartments appear somewhat distinct from each other with no relationships seen between the same inflammatory markers. CF and PCD relationships also differ from each other.

### 3.4 Do inflammatory markers relate to functional or structural lung disease?

Analysis of relationships between *serum* inflammatory markers, FEV_1_%, LCI, and CT scores showed multiple significant correlations in both disease groups. For LCI and CT scores correlations were positive ie. higher values, worse disease and *vice versa* for FEV_1_ (Tables 2 and E6). Notably however, with only one exception (IgG and airway wall thickness), there was no overlap between the relationships demonstrated in CF and those in PCD. Analysis of *sputum* (Tables 3 and E7) was notable in four regards: (i) relationships with physiological measures were largely absent; (ii) several positive correlations were observed with CT scores, indicative of increased inflammation being associated with worse structural lung abnormalities. These correlations, most notably with NE, were exclusive to patients with PCD; (iii) one CT feature, the percentage of air trapping, was highly significantly negatively related to PCD inflammation (IL‐1 β, total cell count); (iv) the only airway inflammatory marker observed to correlate with CT scores in CF was IL‐6 (total score, bronchiectasis severity and wall thickening, the latter *p* < 0.01); however, all relationships, both those non‐significant and significant, were negative (i.e., high IL‐6, better CT scores). Interestingly, although none were statistically significant, the PCD group's CT scores showed a trend towards negative correlation coefficients with markers measured in sputum (Tables 3 and E7).

**Table 2:** Selected values of correlations of inflammatory markers measured in serum with CT scores and lung function.

	IL‐6 serum		IL‐8 serum		IgG		
	*r*	*p*	*r*	*p*	*r*	*p*	
FEV_1_%	−0.26	ns	0.25	ns	−0.24	ns	CF
	a		−0.11	ns	0.23	ns	PCD
LCI	0.13	ns	−0.26	ns	−0.60	ns	CF
	a		−0.09	ns	−0.13	ns	PCD
CT total score	−0.42	ns	−0.30	ns	0.44	ns	CF
	a		0.17	ns	0.48	0.047	PCD
CT severity Bx	0.47	0.036	−0.32	ns	0.54	0.011	CF
	a		0.02	ns	0.36	ns	PCD
CT wall thickness	0.74	< 0.001	−0.09	ns	0.55	0.009	CF
	a		0.52	0.015	0.44	0.042	PCD
CT small plugs	0.22	ns	−0.07	ns	−0.09	ns	CF
	a		0.00	ns	0.43	ns	PCD
CT large plugs	0.43	ns	−0.37	ns	0.33	ns	CF
	a		0.18	ns	0.22	ns	PCD
CT air trapping	−0.23	ns	0.02	ns	−0.02	ns	CF
	a		−0.00	ns	−0.03	ns	PCD

*Note:* Spearman correlation coefficient (*r*) and statistically significant *p*‐value (*p*) is given ns, not significant.

^a^ Not measurable.

**Table 3:** Selected values of correlations of inflammatory markers measured in sputum with markers measured in serum.

	IL‐6 sputum		IL‐8 sputum		NE sputum		
	*r*	*p*	*r*	*p*	*r*	*p*	
FEV_1_%	0.24	ns	−0.03	ns	0.11	ns	CF
	0.06	ns	0.40	ns	0.28	ns	PCD
LCI	−0.07	ns	−0.21	ns	−0.28	ns	CF
	−0.09	ns	0.10	ns	0.17	ns	PCD
CT total score	−0.51	0.024	0.02	ns	0.11	ns	CF
	−0.33	ns	0.40	ns	0.80	< 0.001	PCD
CT total Bx score	−0.34	ns	0.03	ns	0.05	ns	CF
	−0.19	ns	0.38	ns	0.81	< 0.001	PCD
CT extent Bx	−0.15	ns	0.01	ns	0.05	ns	CF
	−0.18	ns	0.41	ns	0.81	< 0.001	PCD
CT severity Bx	−0.52	0.017	0.04	ns	0.05	ns	CF
	−0.20	ns	0.35	ns	0.79	< 0.001	PCD
CT wall thickness	−0.61	0.004	0.09	ns	0.15	ns	CF
	−0.43	ns	−0.08	ns	0.53	0.13	PCD
CT small plugs	−0.42	ns	−0.14	ns	0.00	ns	CF
	−0.15	ns	0.20	ns	0.77	< 0.001	PCD
CT large plugs	−0.42	ns	0.10	ns	0.23	ns	CF
	−0.34	ns	−0.30	ns	0.54	0.001	PCD
CT air trapping	−0.33	ns	−0.30	ns	−0.29	ns	CF
	−0.16	ns	−0.43	ns	0.00	ns	PCD

*Note:* Spearman correlation coefficient (*r*) and statistically significant *p*‐value (*p*) is given; ns, not significant.

### 3.5 Long‐Term Relationships

We assessed the relationship between baseline inflammation and data collected 10 years later. In the two disease groups, gender distribution remained roughly balanced; median FEV_1_ was 53% in CF and 71% in PCD (ns). At the time of data collection, none of the CF patients were receiving highly effective modulator therapy. Demographic data are shown in Table E9.

Due to the small sample size, we used a paired *T*‐test to compare 2009 with 2019. In CF (*n* = 14), median FEV_1_% declined from 67.3 to 53 (range 28.0–98.0, ns), whereas in PCD (*n* = 13), sustained stable lung function was shown over the observation period (median FEV_1_ 68% in 2009 and 71% in 2019 [range 35.0–104.1, ns]). Figure E1 shows the absolute change in FEV₁% compared to baseline FEV1% for both disease groups, with trends over the 10‐year period, although no statistically significant changes were observed in either group.

Correlation analysis between inflammatory markers at baseline and *lung function* parameters in 10 years later was performed. In CF, multiple inflammatory markers (serum IL‐6, CRP, IgG and sputum calprotectin) correlated significantly with later lung function (i.e., high inflammation, low lung function; this was strongest for IL‐6 serum with percentage forced vital capacity (FVC%): *r* = −0.78, *p* < 0.01). Sputum IL‐6 in CF stood out again: higher baseline levels correlated significantly with better FEV_1_% 10 years later (*r* = 0.58, *p* < 0.05). In contrast, high sputum IL‐6 in PCD correlated with worse FEV_1_% and FVC% (*r* = −0.6, *p* < 0.05 and *r* = −0.81, *p* = 0.001); no other relationships were observed in PCD (Table 4).

**Table 4:** Correlation of inflammatory marker measured in 2009 with lung function in 2019.

Parameter	FEV_1_% 2019		FVC% 2019		
	Spearman's *r*	*p* value	Spearman's *r*	*p* value	
IL 6 serum 2009	−0.58	0.035	−0.78	0.002	CF
	a		a		PCD
IL 8 serum 2009	−0.15	ns	−0.11	ns	CF
	0.04	ns	0.16	ns	PCD
Calprotectin serum 2009	0.19	ns	0.28	ns	CF
	0.38	ns	0.23	ns	PCD
CRP serum 2009	−0.65	0.012	−0.60	0.022	CF
	−0.24	ns	−0.00	ns	PCD
IgG serum 2009	−0.38	ns	−0.57	0.032	CF
	0.31	ns	0.06	ns	PCD
IL‐6 sputum 2009	0.58	0.029	0.48	ns	CF
	−0.60	0.028	−0.81	0.001	PCD
IL‐8 sputum 2009	0.39	ns	0.31	ns	CF
	0.35	ns	0.13	ns	PCD
Calprotectin sputum 2009	−0.55	ns	−0.60	0.037	CF
	0.12	ns	−0.32	ns	PCD
IL‐1β sputum 2009	−0.20	ns	−0.19	ns	CF
	0.17	ns	−0.04	ns	PCD
NE sputum 2009	−0.26	ns	−0.27	ns	CF
	0.48	ns	0.44	ns	PCD

*Note:* Spearman correlation coefficient (*r*) and statistically significant *p*‐value (*p*) is given.

^a^ Not measurable.

## 4 Discussion

In the first part of this study, we compared systemic and local inflammatory markers in patients with CF and PCD in a cross‐sectional format. The second part consisted of long‐term observation using real‐world data.

Comparing systemic inflammatory markers at baseline between CF and PCD showed distinct differences, with significantly higher values in CF patients. This is consistent with the assumption that CF is a multi‐system disease with inflammatory processes affecting multiple organ systems. Two well‐known pro‐inflammatory markers were found to be significantly higher in PCD when measured in sputum: IL‐6 and IL‐8. This is consistent with findings of previous studies [9, 10]. However, in those studies, markers were measured during pulmonary exacerbation and not during clinical stability [10]. Another older study compared people with CF to patients with non‐CF bronchiectasis and showed higher sputum IL‐6 levels in non‐CF patients whereas sputum IL‐8 appeared to be higher in CF. Correlations with clinical outcomes were not observed [16].

There are numerous studies investigating inflammation in CF and it has been recognized as a hallmark of the disease [8]. Much less research has been carried out in PCD and little is known about the relationship between inflammatory markers and clinical outcomes. A recent study by Sagel et al. investigated the relationship between sputum biomarkers and lung function in pediatric PCD patients, showing that high sputum NE, IL‐1β, and IL‐8 correlated with worse lung function and chest CT scores at baseline [17]. This is consistent with our results, as NE correlated strongly with almost all features scored in CT at baseline. Our data suggest that sputum IL‐6 may be a long‐term marker in PCD, as high IL‐6 correlated with impaired lung function 10 years later. Taking our results and those of Sagel et al. together, we conclude that local lung inflammation in PCD plays a crucial role regarding long‐term outcome and is not a feature limited to patients with CF.

While there is a consensus that inflammatory processes are the main driver of disease progression in CF, the search for suitable biomarkers has been difficult and results are often conflicting [18]. In our cohort, systemic inflammatory markers were higher in CF patients and more significant correlations with clinical parameters were shown compared to those measured in sputum. At baseline, high IgG, CRP and serum IL‐6 correlated with worse CT scores. These same baseline markers correlated with worse lung function a decade later. The importance of IgG as a long‐term marker is consistent with previous studies [19, 20]. Interestingly, NE did not show long‐term correlations with lung function perhaps related to patients in our cohort being older and NE as a long‐term marker was mainly described in pediatric patients [21, 22, 23]. One possible explanation for the higher systemic inflammatory marker levels observed in CF is the presence of a more pronounced systemic inflammatory burden, potentially related to CFTR dysfunction, and extrapulmonary disease manifestations. In contrast, the higher concentrations of inflammatory markers in sputum from patients with PCD may reflect a predominantly compartmentalized airway inflammation.

Although significantly lower levels of sputum IL‐6 were present compared to PCD, it was the only sputum marker that showed correlations with clinical outcomes in patients with CF. Unexpectedly, high sputum IL‐6 load correlated with better CT scores at baseline and better lung function 10 years later. Sputum IL‐6 has also been described in other diseases such as asthma and obesity, in association with lower lung function and more frequent asthma exacerbation rates [24]. However, its role in CF appears to be different. Low levels of IL‐6 in CF sputum have also been confirmed in other studies raising the possibility that degradation of IL‐6 differs from that of other cytokines [14, 25]. Our data also indicate a different pathophysiological pattern between sputum and serum IL‐6, as only the latter showed high values and long‐term correlations with disease severity. IL‐6 has been primarily discussed as a pro‐inflammatory mediator in CF; however, via classic signaling through the membrane‐bound IL‐6 receptor, it can ultimately promote IL‐10 production, thereby limiting excessive pro‐inflammatory cytokine responses [26]. We are not the first to report an association between high sputum IL‐6 and better lung function. Nixon et al. previously reported on sputum IL‐6 concentrations in patients with chronic pulmonary infection with *P. aeruginosa*, showing a positive correlation between IL‐6 and spirometry and high concentrations of this cytokine after antibiotic treatment [26]. They hypothesized that these findings may reflect changes in the cytokine network that regulates IL‐6 secretion locally [27]. The PROMISE‐Inflammation sub‐study analyzed sputum cytokine profiles in 223 patients with CF before and after initiation of elexacaftor/tezacaftor/ivacaftor (ETI) therapy and reported findings concordant with our results. Specifically, sputum IL‐6 concentrations were lower in patients infected with *P. aeruginosa* and in those with reduced FEV₁, and IL‐6 levels increased after 2 years of ETI treatment while other inflammatory markers declined [28]. A recent study investigated the role of azithromycin on macrophages in patients with CF. A significant increase in IL‐6 and IL‐10 in M1 macrophages following azithromycin treatment was observed leading to a promotion of bacterial phagocytosis. The cytokines IL‐6 and IL‐10 were thus found to have anti‐inflammatory properties in the case of azithromycin therapy [29]. In our cohort, 76% of patients received this therapy and median sputum IL‐6 levels were higher in this group compared to non‐azithromycin therapy (18.6 vs. 7.1 pg/mL). However, numbers were too small for further statistical analysis. In summary, elevated sputum IL‐6 in the context of CF may indicate improved epithelial and immunoregulatory function, favoring regulated inflammatory responses. In contrast, sputum IL‐6 in patients with PCD is more likely to reflect ongoing pro‐inflammatory signaling associated with active infection and tissue injury.

Limitations of this study include the small sample size, especially regarding long‐term follow up and the absence of correction for multiple comparisons in the correlation analysis. However, this was intentionally omitted to preserve the exploratory and hypothesis‐generating nature of the study. This study primarily addresses adult patients, and inflammatory processes in the pediatric population are not fully represented. Additionally, our results reflect the inflammatory status in CF prior modulator therapy, they do not account for potential changes induced by modulator therapy or how these changes may further influence differences in inflammatory processes in patients with CF and PCD. Ultimately, the real‐world nature of the study at the follow‐up time point precludes longitudinal analyzes of systemic inflammatory markers, and therefore correlations between systemic inflammation and lung function cannot be demonstrated.

The strengths of the study include the long follow‐up period of 10 years in both disease groups. Additionally, the disease groups were well matched—intentionally for sex and age, and unintentionally for ppFEV₁—and were all treated at the same centre.

In conclusion, our study emphasizes the fundamental role of inflammatory processes in both diseases but also highlights how they differ. We were able to identify markers that correlated with long‐term deterioration of lung function in PCD (NE, sputum IL‐6) and in CF (IgG, CRP, and serum IL‐6). However, the most striking finding was the correlation between high IL‐6 levels and better CT scores at baseline as well as improved lung function 10 years later. This raises the possibility that IL‐6 may be a protective cytokine in this condition, or associated with a parallel protective mechanism. In the future, further exploration of this pathway may lead to new therapeutic approaches.

## Author Contributions

**Teresa Fuchs:** conceptualization, writing – original draft, resources, formal analysis. **Jacqueline Donovan:** conceptualization, funding acquisition, methodology, writing – review and editing. **Andrew Ives:** writing – review and editing. **Samantha Irving:** writing – review and editing. **Gwyneth Davies:** writing – review and editing. **Claire Hogg:** writing – review and editing. **Eric Alton:** writing – review and editing. **Gemma Wilson:** writing – review and editing. **Andrew Bush:** writing – review and editing. **Jane C Davies:** conceptualization, writing – review and editing, supervision.

## Consent

All persons involved had provided their informed consent prior to inclusion in the study. The study was approved by the Kings College Hospital Research Ethics Committee.

## Conflicts of Interest

The authors declare no conflicts of interest.

## Supporting information

Supporting File:

## Data Availability

The data that support the findings of this study are available on request from the corresponding author. The data are not publicly available due to privacy or ethical restrictions.
