# Supplementary material for: Function and Structure Relationships With Inflammation Differ in Two Chronic Suppurative Lung Diseases
Source: Pediatr Pulmonol. 2026 May 8;61:e71659. doi: 10.1002/ppul.71659 (PMC13155176; doi:10.1002/ppul.71659)
Supplement: Supplementary file 1 — Supporting File: [file PPUL-61-0-s001.docx]

# Methods

Cytokine analysis

Measurement of systemic markers included white cell count (WCC), neutrophils, c-reactive protein (CRP), Immunoglobulin G (IgG), serum calprotectin, and the serum interleukins (IL) 6 and 8. WCC, neutrophils, IgG and CRP were measured within clinical routine in the local lab. IL-6 was measured using Bioplex cytokine assay reagents (Bio-Rad Laboratories, Hemel Hempstead, Hertfordshire UK) analysed on a Luminex 100 analyser (Luminex Corporation, Oosterhout, The Netherlands), following manufacturer’s instructions. A minimum of 100 of each cytokine bead was detected per sample. Serum IL-8 assays were performed by research technicians in Edinburgh, using a commercial kit (KHC0083, Biosource, Invitrogen, CA, USA).

In sputum analysis of total cell count, IL-6, 8, 1β, NE, and sputum calprotectin was performed. All samples, either spontaneous or induced, were combined. Sputum was expectorated into collection pots, placed on ice for transport to the laboratory, and processed within 2 hours before being frozen. Prior to cytokine analysis at a later time point, the samples were completely thawed. Visible plugs were separated from saliva and then divided into portions for the different assays. Sputum was processed using dithiothreitol and Dulbecco's phosphate-buffered saline following the procedure described by Pavord et al. (1). Samples were filtered through a 45 μm pore nylon gauze. The filtrate was then centrifuged at 4 °C for 10 minutes at 800 g. The supernatant was aliquoted and frozen at -80 °C until further analysis; for aliquots used for sputum cytokine analysis, protease inhibitor cocktail was added to the aliquot prior to freezing. The cell pellet was resuspended in 2 ml phosphate-buffered saline and total and viable cell count were performed. IL-8 was again measured by research technicians in Edinburgh using the same kit as for serum IL-8 (KHC0083, Biosource, Invitrogen, CA, USA). Bioplex (Bio-rad,

Hemel Hempstead, UK), was used for analysis of IL-1β and IL-6.

Sputum and serum calprotectin was measured by an in-house ELISA assay performed by research technicians in Edinburgh. Sputum NE activity test was measured by the method described by Suri et al (2).

Assessment of structural and functional lung disease

Spirometry was performed according to the American Thoracic Society and European Respiratory Society recommendations (3). Multiple breath washout was carried out with sulfur hexafluoride as tracer gas. Lung Clearance Index (LCI) is given as the mean value of at least two reproducible repetitions from washouts of satisfactory quality. Chest CT imaging was scored on a lobar basis by two independent radiologists using a grading system based on Roberts *et al* (4). In addition to an overall score and total bronchiectasis score, the following features were assessed in more detail: extent of bronchiectasis, wall thickness, small and large plugs as well as air trapping. The scores of both observers were added together, resulting in a range of 12 to 84 for each CT characteristic. The final score is expressed as a percentage of the maximum possible score for that characteristic.

**Table E1:** Comparison of systemic inflammatory markers between the disease groups at baseline (2009). Data is presented as median (range). Lymphocytes, eosinophils, basophils, IL-1β, IL-10, IL-12, and TNFα were also measured but did not show significant differences or were not detectable.

| **Variable** | **CF** | **PCD** | **p-value** |
| --- | --- | --- | --- |
| WCC (10^9^/L) | 9.4 (4.8-15) | 7.3 (4.1-13.4) | 0.054^2^ |
| Neutrophils (10^9^/L) | 6.4 (2.5-10.5) | 4.2 (2-9.5) | 0.024^2^ |
| CRP (mg/dl) | 2 (0.5-24) | 2 (0.5-28) | 0.464^1^ |
| IgG (g/l) | 13.6 (6.7-25.5) | 10 (4.7-14.8) | 0.054^1^ |
| Calprotectin (mg/l) | 15 (5.8-52) | 11.4 (2.5-49.0) | 0.032^1^ |
| IL-8 (pg/ml) | 3.1 (1.7-40.3) | 1.8 (0.7-4.1) | <0.006^1^ |
| IL-6 (pg/ml) | 7.2 (7.2-15.5) | <7.2 | <0.006^1^ |

*Legend:* ^1^ Mann Whitney Test, ^2^ unpaired T-Test, values were corrected for multiple comparisons using Bonferoni correction.

# **Table E2:** Comparison of sputum inflammatory markers between the disease groups at baseline (2009). Data is presented as median (range). Absolute neutrophils, TNFα, and MPO were also measured but did not show significant differences or were not detectable.

| **Variable** | **CF** | **PCD** | **p-value** |
| --- | --- | --- | --- |
| IL-1β (pg/ml) | 470 (50.5-2873.8) | 163.6 (11.5-2385.8) | 0.055^1^ |
| Total cell count (10^6^/g) | 10.9 (0.5-76) | 6.7 (0.7-64) | 0.359^1^ |
| Neutrophil Elastase (mU) | 537 (32-1894) | 537 (32-1389) | 0.44^2^ |
| Calprotectin (μg/ml) | 816.8 (200.2-3463) | 935 (9.2-3844.7) | 0.979^1^ |
| IL-6 (pg/ml) | 10.9 (4.6-270.7) | 86 (14.8-1319.6) | <0.005^1^ |
| IL-8 (pg/ml) | 9218.6 (2399.7-23922.6) | 26251.4 (3247.3-74500) | <0.005^1^ |

*Legend:* ^1^ Mann Whitney Test, ^2^ unpaired T-Test, values were corrected for multiple comparisons using Bonferoni correction.

# **Table E3:** Correlations of inflammatory markers measured in serum with each other**.** Spearman correlation coefficient (r) and statistically significant p-value (p) is given, with CF shown in red and PCD in blue**.**

|  | **IL-6 serum** | | **IL-8 serum** | | **Calprotectin serum** | | **IgG** | | **CRP** | | **White blood count** | | **Neutrophil count** | |  |
| --- | --- | --- | --- | --- | --- | --- | --- | --- | --- | --- | --- | --- | --- | --- | --- |
|  | **r** | **p** | **r** | **p** | **r** | **p** | **r** | **p** | **r** | **p** | **r** | **p** | **r** | **p** |  |
| **IL-6 serum** |  | | -0.20  a | ns | -0.01  a | ns | 0.62  a | 0.003 | 0.47  a | 0.035 | 0.34  a | ns | 0.38  a | ns | CF  PCD |
| **IL-8 serum** | - | |  | | -0.06  0.03 | ns  ns | 0.06  0.41 | ns  ns | -0.13  -0.14 | ns  ns | -0.24  -0.36 | ns  ns | -0.35  -0.27 | ns  ns | CF  PCD |
| **Calprotectin serum** | - | | - | |  | | -0.07  0.32 | ns  ns | -0.08  0.32 | ns  ns | 0.50  0.51 | 0.022  0.016 | 0.39  0.72 | ns  <0.001 | CF  PCD |
| **IgG** | - | | - | | - | |  | | 0.10  0.21 | ns  ns | 0.11  0.21 | ns  ns | 0.09  0.25 | ns  ns | CF  PCD |
| **CRP** | - | | - | | - | | - | |  | | -0.05  0.41 | ns  ns | 0.05  0.50 | ns  0.019 | CF  PCD |
| **White blood count** | - | | - | | - | | - | | - | |  | | 0.92  0.90 | <0.001  <0.001 | CF  PCD |

*Legend*: a = not measurable, ns= not significant

# **Table E4:** Correlations of inflammatory markers measured in sputum with each other**.** Spearman correlation coefficient (r) and statistically significant p-value (p) is given, with CF shown in red and PCD in blue**.**

|  | **IL-8 sputum** | | **IL-1β sputum** | | **NE sputum** | | **Calprotectin sputum** | | **Total cell count sputum** | |  |
| --- | --- | --- | --- | --- | --- | --- | --- | --- | --- | --- | --- |
|  | **r** | **p** | **r** | **p** | **r** | **p** | **r** | **p** | **r** | **p** |  |
| **IL-6 sputum** | 0.26  0.02 | ns  ns | -0.29  0.08 | ns  ns | -0.10  -0.32 | ns  ns | -0.24  0.22 | ns  ns | -0.03  -0.02 | ns  ns | CF  PCD |
| **IL-8 sputum** |  | | 0.47  0.79 | 0.039  <0.001 | 0.53  0.51 | 0.018  0.017 | 0.22  0.24 | ns  ns | 0.08  0.65 | ns  0.001 | CF  PCD |
| **IL-1β sputum** | - | |  | | 0.45  0.17 | 0.037  ns | 0.61  0.04 | 0.005  ns | 0.416  0.76 | ns  <0.001 | CF  PCD |
| **NE sputum** | - | | - | |  | | 0.61  0.48 | 0.005  0.027 | 0.635  0.41 | 0.002  ns | CF  PCD |
| **Calprotectin**  **sputum** | - | | - | | - | |  | | 0.651  0.30 | 0.003  ns | CF  PCD |
| **Total cell count sputum** | - | | - | | - | | - | |  | |  |

*Legend*: ns= not significant

# **Table E5:** Correlations of inflammatory markers measured in sputum with markers measured in serum**.** Spearman correlation coefficient (r) and statistically significant p-value (p) is given, with CF shown in red and PCD in blue**.**

|  | **IL-6 serum** | | **IL-8 serum** | | **Calprotectin serum** | | **IgG** | | **CRP** | | **White cell count** | |  |
| --- | --- | --- | --- | --- | --- | --- | --- | --- | --- | --- | --- | --- | --- |
|  | **r** | **p** | **r** | **p** | **r** | **p** | **r** | **p** | **r** | **p** | **r** | **p** |  |
| **IL-6 sputum** | -0.22  a | ns | -0.07  -0.40 | ns  ns | -0.01  -0.22 | ns  ns | -0.31  -0.21 | ns  ns | -0.22  -0.10 | ns  ns | 0.37  -0.25 | ns  ns | CF  PCD |
| **IL-8 sputum** | 0.33  a | ns | -0.18  -0.38 | ns  ns | 0.15  0.19 | ns  ns | 0.10  0.03 | ns  ns | -0.17  0.21 | ns  ns | 0.61  0.52 | 0.005  0.016 | CF  PCD |
| **IL-1β sputum** | 0.56  a | 0.01 | -0.24  -0.25 | ns  ns | -0.07  -0.07 | ns  ns | 0.34  0.10 | ns  ns | -0.14  -0.04 | ns  ns | 0.19  0.25 | ns  ns | CF  PCD |
| **NE sputum** | 0.14  a | ns | -0.03  0.09 | ns  ns | -0.17  0.57 | ns  0.006 | -0.12  0.39 | ns  ns | -0.33  0.46 | ns  0.035 | 0.01  0.50 | ns  0.020 | CF  PCD |
| **Calprotectin sputum** | 0.02  a | ns | -0.27  -0.13 | ns  ns | -0.84  0.28 | ns  ns | -0.00  0.33 | ns  ns | -0.44  0.23 | ns  ns | 0.05  0.33 | ns  ns | CF  PCD |

*Legend:* a = not measurable, ns= not significant

| **CT large plugs** | 0.43  a | ns | -0.37  0.18 | ns  ns | -0.11  0.53 | ns  0.012 | 0.33  0.22 | ns  ns | 0.19  0.11 | ns  ns | 0.00  0.23 | ns  ns | 0.13  0.37 | ns  ns | CF  PCD |
| --- | --- | --- | --- | --- | --- | --- | --- | --- | --- | --- | --- | --- | --- | --- | --- |
| **CT air trapping** | -0.23  a | ns | 0.02  -0.00 | ns  ns | 0.29  0.30 | ns  ns | -0.02  -0.03 | ns  ns | 0.02  0.32 | ns  ns | -0.25  0.13 | ns  ns | -0.22  0.15 | ns  ns | CF  PCD |

*Legend:* Bx= bronchiectasis, LCI= Lung Clearance Index, CT= computer tomography, a = not measurable, ns= not significant

**Table E6:** Complementary table of correlations of inflammatory markers measured in serum with CT scores and lung function**.** Spearman correlation coefficient (r) and statistically significant p-value (p) is given, with CF shown in red and PCD in blue**.**

|  | **Calprotectin serum** | | **CRP** | | **White cell count** | | **Neutrophils count** | |  |
| --- | --- | --- | --- | --- | --- | --- | --- | --- | --- |
|  | **r** | **p** | **r** | **p** | **r** | **p** | **r** | **p** |  |
| **FEV_1_%** | 0.08  -0.16 | ns  ns | -0.40  0.12 | ns  ns | 0.15  0.09 | ns  ns | -0.17  0.47 | ns  0.038 | CF  PCD |
| **LCI** | 0.26  0.38 | ns  ns | 0.51  0.17 | 0.016  ns | -0.03  0.38 | ns  ns | -0.19  -0.23 | ns  ns | CF  PCD |
| **CT total score** | 0.12  0.64 | ns  0.005 | 0.50  0.31 | 0.024  ns | 0.04  0.33 | ns  ns | 0.12  0.52 | ns  0.033 | CF  PCD |
| **CT severity Bx** | 0.03  0.58 | ns  0.005 | 0.43  0.25 | 0.048  ns | 0.00  0.33 | ns  ns | 0.10  0.52 | ns  0.014 | CF  PCD |
| **CT wall thickness** | 0.18  0.56 | ns  0.007 | 0.50  0.30 | 0.020  ns | 0.18  0.10 | ns  ns | 0.26  0.30 | ns  ns | CF  PCD |
| **CT small plugs** | 0.16  0.71 | ns  <0.001 | 0.40  0.62 | ns  0.002 | -0.10  0.45 | ns  0.037 | -0.15  0.68 | ns  <0.001 | CF  PCD |
| **CT large plugs** | -0.11  0.53 | ns  0.012 | 0.19  0.11 | ns  ns | 0.00  0.23 | ns  ns | 0.13  0.37 | ns  ns | CF  PCD |
| **CT air trapping** | 0.29  0.30 | ns  ns | 0.02  0.32 | ns  ns | -0.25  0.13 | ns  ns | -0.22  0.15 | ns  ns | CF  PCD |

*Legend:* Bx= bronchiectasis, LCI= Lung Clearance Index, CT= computer tomography, a = not measurable, ns= not significant

# **Table E7:** Complementary table of correlations of inflammatory markers measured in sputum with CT scores and lung function**.** Spearman correlation coefficient (r) and statistically significant p-value (p) is given, with CF shown in red and PCD in blue**.**

|  | **IL-1β sputum** | | **Calprotectin sputum** | | **Total cell count sputum** | |
| --- | --- | --- | --- | --- | --- | --- |
|  | **r** | **p** | **r** | **p** | **r** | **p** |
| **FEV_1_%** | -0.22  0.32 | ns  ns | -0.10  0.17 | ns  ns | -0.05  0.45 | ns  0.040 |
| **LCI** | -0.30  -0.001 | ns  ns | -0.13  0.12 | ns  ns | -0.25  -0.18 | ns  ns |
| **CT total score** | 0.12  0.46 | ns  ns | 0.10  0.32 | ns  ns | 0.05  0.48 | ns  0.047 |
| **CT total Bx score** | 0.008  0.23 | ns  ns | 0.09  0.36 | ns  ns | 0.02  0.39 | ns  ns |
| **CT extent Bx** | -0.19  0.25 | ns  ns | -0.05  0.39 | ns  ns | -0.04  0.43 | ns  0.047 |
| **CT severity Bx** | 0.26  0.20 | ns  ns | 0.28  0.32 | ns  ns | 0.11  0.34 | ns  ns |
| **CT wall thickness** | 0.41  -0.11 | ns  ns | 0.18  0.05 | ns  ns | 0.06  0.08 | ns  ns |
| **CT small plugs** | -0.27  -0.10 | ns  ns | -0.16  0.37 | ns  ns | -0.17  0.04 | ns  ns |
| **CT large plugs** | 0.40  -0.24 | ns  ns | 0.17  0.24 | ns  ns | 0.21  -0.11 | ns  ns |
| **CT air trapping** | -0.27  -0.80 | ns <0.001 | -0.11  -0.07 | ns  ns | -0.16  -0.67 | ns  <0.001 |

*Legend:* Bx= bronchiectasis, LCI= Lung Clearance Index, CT= computer tomography, a = not measurable, ns= not significant

# **Table E8:** Lower limits of detection of assays used.

|  | Limit of detection of assay used |
| --- | --- |
| IL-6 serum | 7.2 pg/ml |
| IL-8 serum | 0.7 pg/ml |
| Calprotectin serum | 0.78 mg/L |
| IL-6 sputum | 2.4 pg/ml |
| IL-8 sputum | 0.07 ng/mL |
| IL-1β sputum | 4.4 pg/ml |
| NE sputum | 32 pg/ml |
| Calprotectin  sputum | 7.8 mg/L |

| **Variable** | **CF** | **PCD** | **p-value** |
| --- | --- | --- | --- |
| Number | 14 | 13 |  |
| Age, (y) median (range) | 36 (23-59) | 38 (21-63) | ns |
| Sex, n  Male (%)  Female (%) | 9 (64.3)  5 (35.7) | 9 (69.2)  4 (30.8) |  |
| Lost to follow up, n (%) | 4 (28.6) | 6 (46.2) |  |
| Deceased, n (%) | 2 (14.3) | 2 (15.4) |  |
| FEV_1_%, median (range) | 53.0 (28.0-98.0) | 71.1 (35.0-104.1) | ns |
| FVC% median (range) | 83.0 (32.0-112.4) | 81.7 (49.0-115.4) | ns |

# **Table E9:** Demographic data and differences of study groups at follow up, 2019. Data is presented as median (range) or percentage with statistical analysis by Mann Whitney U Test.

**Figure E1: Absolute change of ppFEV_1_ in 10 years compared to baseline ppFEV_1_ in 2009.**
(a) CF (red squares) and (b) PCD (blue circles). ns=not significant.


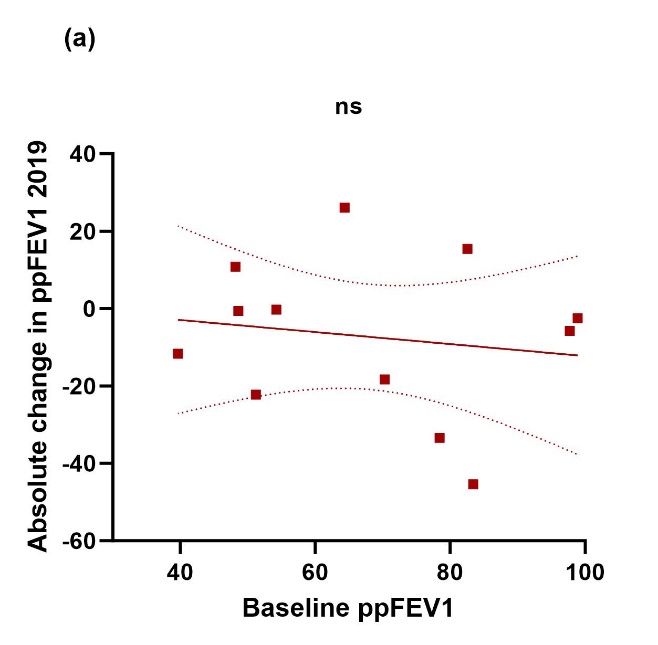

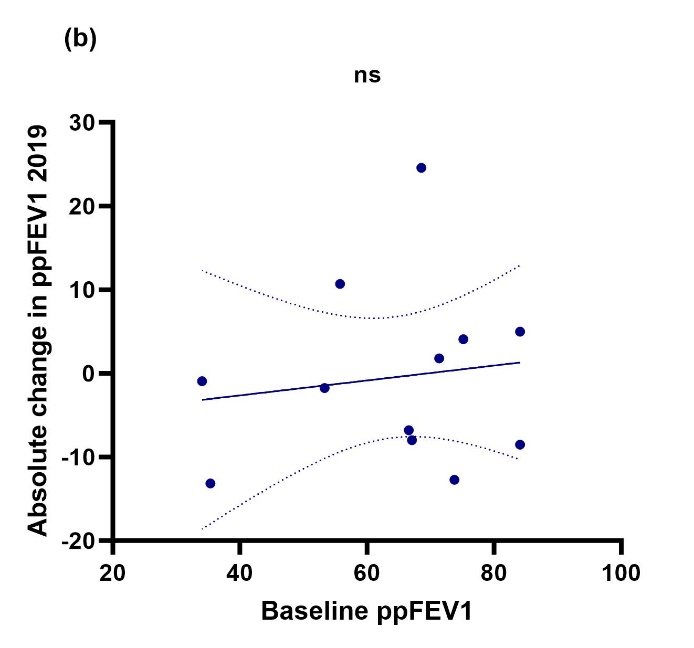


Literature Cited

1. Pavord ID, Pizzichini MM, Pizzichini E, Hargreave FE. The use of induced sputum to investigate airway inflammation. Thorax 1997; 52(6):498–501.

2. Suri R, Marshall LJ, Wallis C, Metcalfe C, Shute JK, Bush A. Safety and use of sputum induction in children with cystic fibrosis. Pediatr Pulmonol 2003; 35(4):309–13.

3. Pellegrino R, Viegi G, Brusasco V, Crapo RO, Burgos F, Casaburi R et al. Interpretative strategies for lung function tests. Eur Respir J 2005; 26(5):948–68.

4. Roberts HR, Wells AU, Milne DG, Rubens MB, Kolbe J, Cole PJ et al. Airflow obstruction in bronchiectasis: correlation between computed tomography features and pulmonary function tests. Thorax 2000; 55(3):198–204.
